# Placenta segmentation redefined: review of deep learning integration of magnetic resonance imaging and ultrasound imaging

**DOI:** 10.1186/s42492-025-00197-8

**Published:** 2025-07-15

**Authors:** Asmaa Jittou, Khalid El Fazazy, Jamal Riffi

**Affiliations:** Laboratory of Computer Science, Innovation, and Artificial Intelligence, Faculty of Science Dhar El Mahraz, University Sidi Mohamed Ben Abdellah, 30000 Fes, Morocco

**Keywords:** Deep learning, Segmentation, Placenta, Magnetic resonance imaging, Ultrasound

## Abstract

Placental segmentation is critical for the quantitative analysis of prenatal imaging applications. However, segmenting the placenta using magnetic resonance imaging (MRI) and ultrasound is challenging because of variations in fetal position, dynamic placental development, and image quality. Most segmentation methods define regions of interest with different shapes and intensities, encompassing the entire placenta or specific structures. Recently, deep learning has emerged as a key approach that offer high segmentation performance across diverse datasets. This review focuses on the recent advances in deep learning techniques for placental segmentation in medical imaging, specifically MRI and ultrasound modalities, and cover studies from 2019 to 2024. This review synthesizes recent research, expand knowledge in this innovative area, and highlight the potential of deep learning approaches to significantly enhance prenatal diagnostics. These findings emphasize the importance of selecting appropriate imaging modalities and model architectures tailored to specific clinical scenarios. In addition, integrating both MRI and ultrasound can enhance segmentation performance by leveraging complementary information. This review also discusses the challenges associated with the high costs and limited availability of advanced imaging technologies. It provides insights into the current state of placental segmentation techniques and their implications for improving maternal and fetal health outcomes, underscoring the transformative impact of deep learning on prenatal diagnostics.

## Introduction

Pregnancy is a remarkable physiological state characterized by the dynamic interplay between mother and fetus, central to the placenta. This organ is pivotal for ensuring the exchange of nutrients, gases, and waste products between maternal and fetal circulation while also secreting hormones critical for maintaining pregnancy and providing immune protection to the fetus [1]. Despite its significance, the accurate assessment of the placenta remains a challenging task, primarily because of its reliance on manual segmentation in medical imaging, a process that is time-consuming, subjective, and prone to interobserver variability. Addressing this challenge is vital because precise placental evaluation is crucial for optimal maternal and fetal health outcomes.

Recent advancements in artificial intelligence (AI), particularly deep learning, have offered transformative solutions to the limitations of manual segmentation. Deep learning models have demonstrated exceptional capabilities in medical imaging analyses, including placental segmentation using magnetic resonance imaging (MRI) and ultrasound. These technologies provide critical benefits like enhanced diagnostic accuracy (ACC), reduced subjectivity, and improved efficiency. For instance, automated segmentation can improve the detection of abnormalities, for example, placenta previa and placenta accreta, allowing timely and informed clinical decision-making. Moreover, these models standardize prenatal care by minimizing operator dependence and variability, making them applicable in diverse clinical settings.

The state of research in this field highlights the potential and limitations of the current methods. Studies underscore the effectiveness of convolutional neural networks (CNNs) and other advanced models in prenatal diagnostics [2–4]. However, challenges persist, i.e., the high cost and limited availability of advanced imaging technologies as well as the need for models tailored to specific imaging modalities and clinical scenarios. Although MRI-based segmentation provides superior detail, ultrasonography remains more accessible and widely used, creating a gap in the integration of these complementary modalities for placental analysis.

This review aims to bridge this gap by exploring the integration of deep learning into placental imaging and focusing on segmentation techniques for MRI and ultrasound modalities. The primary goal is to synthesize recent advancements (2019–2024) used for placenta segmentation, evaluate the strengths and limitations of existing models, and propose future directions for enhancing segmentation performance. Our contribution lies in presenting a comprehensive analysis of deep-learning approaches and their potential to revolutionize prenatal diagnostics, particularly through the integration of complementary imaging modalities.

This review begins with an introduction to placental anatomy, imaging modalities, challenges in placental imaging and abnormalities, and computational challenges in MRI and ultrasound imaging, followed by a discussion of deep learning models for placental segmentation, covering MRI and ultrasound approaches. A comparative analysis evaluates these models by focusing on the U-Net innovation. The Discussion section addresses the clinical implications and challenges, and the Conclusion section summarizes the key findings and transformative impact of deep learning in placental imaging.

### Placenta anatomy

**Fig. 1:**
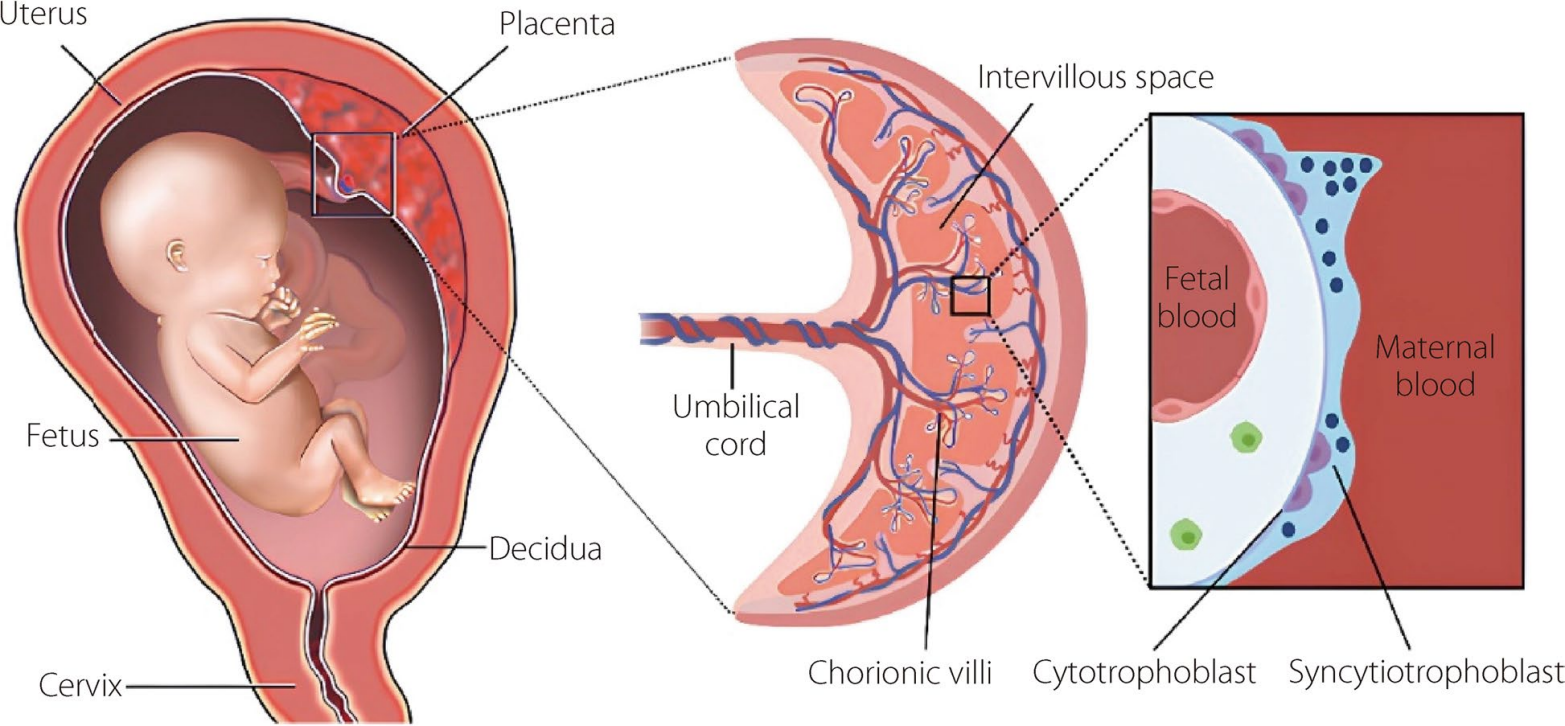
Anatomical structure and function of the placenta during pregnancy

The placenta is a disk-shaped organ with bumpy tissue and a dense network of blood vessels, giving it a dark red appearance at full term. It connects to the baby via the umbilical cord, with blood vessels spreading across the placental disclike branches. The side attached to the uterus is deep reddish-blue, whereas the side facing the baby is grayish (Fig. 1). Structurally, the placenta has two main components: the fetal component, derived from the blastocyst, and the maternal component, derived from the uterine lining (the decidua). Chorionic villi increase the surface area for material exchange between fetal and maternal blood within the intervillous space, where these villi lie in maternal blood, facilitating the exchange of nutrients and waste [5].

**Fig. 2:**
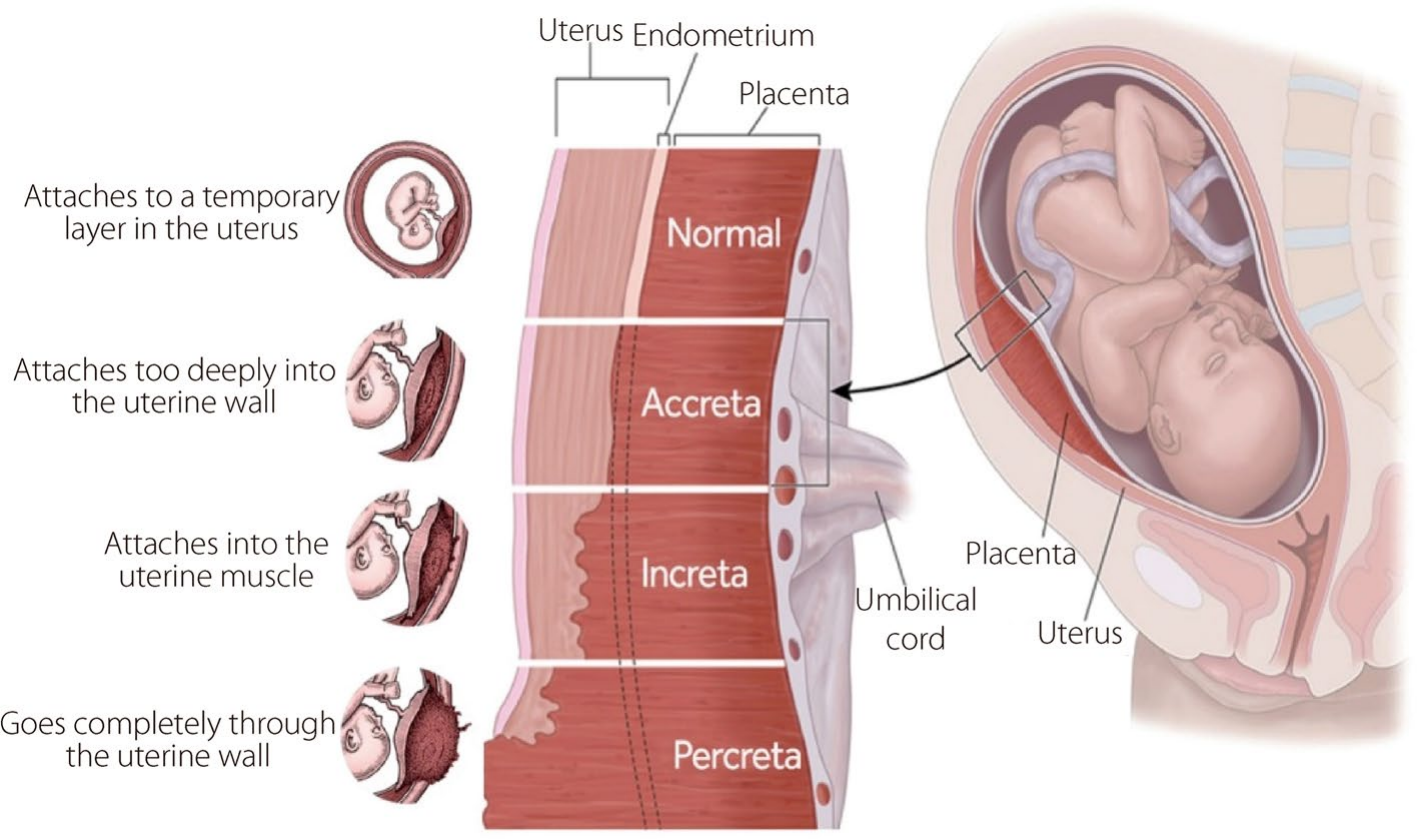
Comparison of placenta attachment types: normal, accreta, increta, and percreta

An intimate connection between the fetal and maternal components of the placenta, facilitated by chorionic villi, is essential for a healthy pregnancy. However, disruptions to this delicate balance, as seen in placental abnormalities including placenta accreta spectrum (PAS), can have significant consequences. In a normal pregnancy, the placenta is implanted in the inner lining of the uterus (endometrium), whereas in PAS, the placenta attaches too deeply and grows into the muscular wall of the uterus (myometrium), which is associated with significantly higher risks for mothers and children at birth. These can range from placenta accreta, in which the chorionic villi attach to the myometrium but do not invade it, to placenta percreta, in which the villi penetrate the myometrium and extend beyond the serosa. Other risk factors for PAS include previous uterine surgery, placenta previa, and an elevated body mass index [6]. Placenta accreta is characterized by the abnormal adherence of chorionic villi to the myometrium without invasion and represents the mildest form of this disorder. In placenta increta, chorionic villi partially invade the myometrium, constituting an intermediate severity condition. The most severe form, placenta percreta, involves chorionic villi that penetrate the entire myometrium and potentially extend beyond the uterine serosa. These conditions pose significant risks during pregnancy and delivery, necessitating careful monitoring and specialized management to prevent severe complications (Fig. 2).

### Imaging modalities

Developments in placental imaging technologies from traditional ultrasound to advanced MRI and even experimental photoacoustic imaging, have significantly expanded our understanding of placental functions and what may be considered healthy levels of operation. Imaging techniques, including ultrasound and MRI, are critical for assessing placental health and location. Ultrasound is an invaluable tool because it is both safe and affordable and provides essential information on the development and localization of the placenta, which are key factors in successful pregnancy outcomes. MRI is reserved for more complex imaging needs, especially when ultrasound results are inconclusive. It offers very high spatial and contrast resolutions, which are crucial for detailed placental evaluation without the risks associated with ionizing radiation [7]. Both ultrasound and MRI are indispensable in fetal imaging, and each plays a critical role in managing pregnancy and diagnosing fetal conditions.

Ultrasonography, which is often the first choice for prenatal care, is widely preferred because of its safety, cost-effectiveness, and real-time imaging capabilities. It excels in routine evaluations and follow-up throughout pregnancy. Ultrasound technology has evolved significantly, enhancing placental segmentation through various methods like color Doppler, three-dimensional ultrasound (3DUS), and the incorporation of advanced software algorithms. Traditional two-dimensional ultrasound (2DUS) provides basic morphological information, whereas 3DUS and four-dimensional ultrasound techniques provide volumetric measurements that are crucial for detailed placental analysis. These techniques have expanded their diagnostic power by providing detailed anatomical views and dynamic assessments of the fetus and placenta [8, 9].

MRI**,** with its superior soft-tissue contrast, is crucial when ultrasound reaches its limits. It is particularly useful for diagnosing the central nervous system and complex syndromes. MRI is safe during pregnancy, is free from ionizing radiation and offers detailed images of fetal brain development. Doctors often recommend it when ultrasound results are inconclusive or show abnormalities, thus enhancing diagnostic certainty and aiding in complex pregnancy management [10, 11].

The synergistic use of ultrasound and MRI enhances diagnostic ACC, particularly for conditions notably exemplified by placenta previa and placenta accreta. This combined approach improves diagnostic ACC (86.27%) and sensitivity (97.78%) and reduces false negatives (72.00%) compared to using either method alone [12]. It provides a comprehensive evaluation of the placental location and invasion depth, which are crucial for planning safe delivery strategies to prevent severe complications, particularly hemorrhage. Fetal MRI complements ultrasound by offering detailed evaluations of the placenta and umbilical cord, especially in complex cases where high-resolution imaging is essential for guiding clinical management and improving pregnancy outcomes [7, 13]. Additionally, MRI effectively identifies conditions, i.e., brain anomalies, lung pathologies, and reductions in amniotic fluid, which are often undetectable by ultrasound [14].

### Challenges in placental imaging and placental abnormalities

Annotated placental datasets are often limited by their small size and imbalance, primarily because of the time-intensive nature of manual labeling and ethical constraints on data sharing. These datasets frequently underrepresent certain placental abnormalities or developmental stages, notably rare pathologies or early developmental periods, leading to challenges in training robust and generalizable deep learning models [15, 16]. Manual labeling requires expert knowledge and significant resources, whereas strict ethical regulations further constrain data availability [17, 18]. Consequently, these limitations result in models that are prone to overfitting, where performance on training data is strong, but generalization to unseen data is poor [19, 20]. Perspectives on placental datasets highlight the complexity of placental phenotypic changes and their multi-factorial etiology, which further complicates efforts to build large, diverse datasets. Additionally, placental research is hindered by technological limitations in imaging and bioengineering, which restrict the comprehensive quantitative assessment of structural growth [21]. To address these issues, researchers have adopted strategies including data augmentation-like rotation and flipping techniques-and the use of generative adversarial networks to create synthetic data [17, 18]. Moreover, virus-like particles and nanoparticles have been explored for their potential in targeted drug delivery, demonstrating the importance of advanced computational techniques to improve placental data collection and modeling. Integrating public datasets and leveraging automated annotation tools can enhance dataset size and diversity while reducing manual effort [20, 22].

**Fig. 3:**
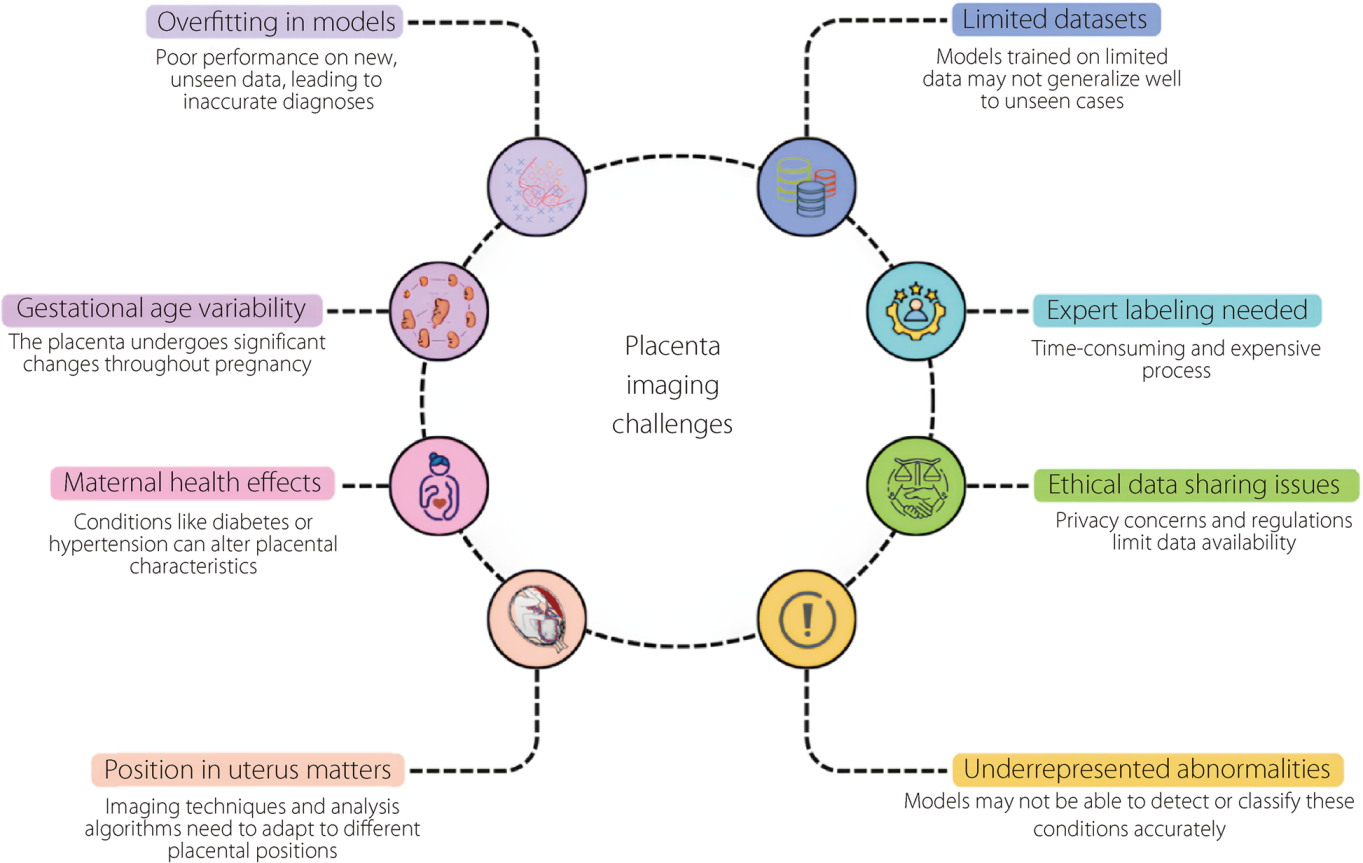
Overview of challenges in placental imaging

Moreover, placental appearance is significantly influenced by gestational age, position, and maternal health conditions, which collectively complicate model training for medical imaging and reduce segmentation ACC, particularly in ultrasound imaging. As the pregnancy progresses, the placenta undergoes notable morphological changes, transitioning from a relatively uniform discoid shape to more complex structures characterized by lobulation and varying thicknesses, typically ranging from 2 to 4 cm. These changes, along with other factors, present significant imaging challenges (Fig. 3). Maternal diabetes or infections can lead to abnormal thickening or thinning [23]. The position of the placenta within the uterus, whether anterior, posterior, lateral, or fundal, also affects its visual characteristics; for example, anterior placentas provide better contrast on ultrasound, whereas posterior placentas may be obscured by the fetus, leading to shadowing effects [24]. Moreover, health conditions, e.g., placenta previa, can cause the placenta to partially or completely cover the cervix, and other abnormalities, such as bilobed or succenturiate lobes, introduce further complexity into imaging and modeling efforts, affecting clinical assessments and fetal development. These variabilities necessitate the incorporation of diverse placental appearances into training datasets for machine learning models to enhance their robustness and ACC in segmentation tasks [25]. Consequently, addressing these challenges requires advanced imaging techniques and sophisticated algorithms capable of adapting to the wide range of placental morphologies encountered in clinical practice.

The subsequent sections provide a concise overview of placental anatomy and function. We then delve into the latest technological advancements in placental imaging, with a specific focus on deep learning models for placenta segmentation. This review explores the clinical implications of these advancements and their potential to improve maternal and fetal health outcomes. Finally, we discuss the transformative impact of deep learning on prenatal diagnostics and highlight areas for future research.

### Computational challenges in MRI and ultrasound imaging

Given the computational challenges associated with processing large MRI volumes and the intensive preprocessing required for ultrasound, parallel processing techniques present a promising solution for mitigating these burdens. MRI datasets often consist of high-dimensional volumetric data, and deep learning models for segmentation, reconstruction, or classification require extensive computational power. Traditional CPU-based processing is limited in its ability to handle such workloads efficiently owing to memory bandwidth constraints and sequential execution limitations. Unlike traditional systems, parallel architectures can perform many tasks at the same time, which makes them much faster. GPUs, which were first made for graphics, are now used to speed up tasks like image processing and scientific calculations. FPGAs are special chips that can be programmed for specific jobs, making them efficient and powerful for real-time tasks. Because both GPUs and FPGAs can run multiple computations at once, they help reduce the time needed for training and running models. Parallelized lattice Boltzmann models can efficiently handle large-scale medical simulations, demonstrating scalability across high-performance computing (HPC) platforms, i.e., advanced research computing high end resource, a HPC facility that supports various scientific fields, and Blue Waters, one of the most powerful supercomputers for open scientific research worldwide and funded by the National Science Foundation [26]. These platforms distribute computational tasks across thousands of cores, enabling near-real-time performance even when processing large MRI volumes.

Furthermore, heterogeneous system-on-chip (SoC) implementations have been explored to optimize MRI processing, particularly for real-time hemodynamic simulations. The HemeLB framework, a massively parallel solver for simulating cerebral blood flow, has been deployed on various hardware accelerators, including FPGAs and embedded GPU platforms, to enhance computational efficiency [27]​. Compared to CPU-only implementations, FPGA-based architectures provide improved energy efficiency and lower latency, making them suitable for clinical applications where the computational overhead must be minimized. In particular, the Zynq SoC-based acceleration of the lattice Boltzmann method has demonstrated a processing speedup of up to a factor of 52 compared with traditional dual-core ARM (Advanced RISC machines) implementations, demonstrating the effectiveness of parallel architectures in medical imaging tasks [28]​.

For ultrasound imaging, preprocessing steps such as noise suppression and artifact removal require additional computational resources. Parallel computing approaches have been widely adopted to accelerate these processes, allowing real-time denoising and edge enhancement. HPC frameworks, including compute unified device architecture (CUDA)-based implementations, enable the efficient execution of advanced filtering algorithms directly on GPUs, significantly reducing processing latency. Research has indicated that integrating parallel computing techniques into ultrasound imaging pipelines can enhance real-time visualization capabilities, allowing immediate feedback during clinical assessments [26]. In addition, CUDA-based rendering techniques facilitate real-time interactive visualization, which is a crucial feature in applications notably fetal imaging and intraoperative ultrasound guidance [28]​​.

Another critical advantage of parallel computing in medical imaging is its ability to perform real-time segmentation and classification of ultrasound and MRI data. Deep learning models, including CNNs and transformer-based architectures, require extensive matrix multi-plication, which is inherently parallelizable. By leveraging GPU-accelerated tensor processing, these models can efficiently analyze volumetric data in real-time, making automated diagnosis and anomaly detection feasible within clinical workflows. Hybrid CPU-GPU implementations further improve performance by offloading memory-intensive operations to high-speed GPU memory while maintaining control logic on the CPU [27]​.

In summary, the integration of parallel processing methodologies into medical imaging workflows can address the computational limitations of MRI and ultrasound, thereby making real-time applications more feasible in clinical settings. By leveraging multi-core architectures, high-performance GPUs, and distributed computing frameworks, medical imaging can achieve faster and more accurate diagnostics and ultimately improve patient outcomes. Advancements in parallel computing, from high-performance supercomputers to embedded heterogeneous architectures, have demonstrated significant potential for overcoming the real-time processing challenges associated with deep-learning-based medical imaging analysis.

### Deep learning models for placenta segmentation

Deep learning algorithms are increasingly central to processing ultrasound images of fetuses and are used to detect standard planes, analyze anatomical structures, and estimate biometric parameters [29]. As these technologies advance, the integration of AI with both MRI and ultrasound has significantly enhanced prenatal diagnostics, revolutionizing the field of medical imaging. Their ability to learn complex patterns and features directly from data provides fresh insights into fetal health. This convergence potentially leads to earlier detection of developmental issues and enables more customized prenatal care.

### MRI-based models

The evolution of MRI-based placental segmentation methodologies has seen significant advancements through the adoption of sophisticated deep learning architectures, each tailored to address specific challenges in the segmentation process. Table 1 presents a comparative visualization of the segmentation results from various MRI-based models, showing the ground truth *vs* the predicted outcomes from several key studies [30–34].

**Table 1:**
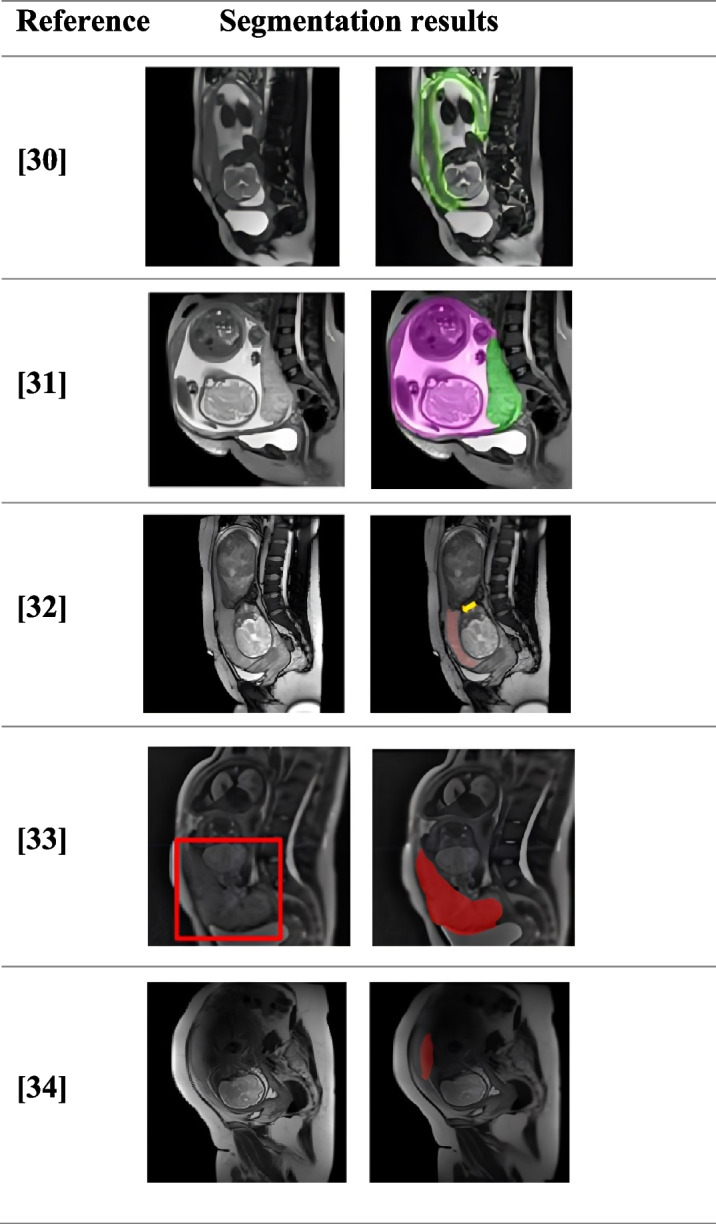
Placenta segmentation results from related works using MRI: ground truth and predicted outcomes

Li et al. [30] demonstrated the refinement fusion U-Net (RFU-Net) enhances placenta segmentation in MRI scans by integrating specialized modules with the standard U-Net architecture [35] to effectively handle variations in placental position and shape. The study included gestational ages ranging from 28 to 40 weeks, involving 2D MRI images from 140 cases (70 normal, 70 PAS) for training and separate validation and test sets comprising both normal and PAS cases. The model includes the following: (1) the fusion multi-scale feature (FMF) module, which enhances image segmentation by systematically extracting and integrating features across multiple scales, as observed in its application for lesion segmentation [36] and MRI analysis [37]; and (2) the refinement segmentation module (RSM) for fine-tuning segmentation results, providing detailed correction and refinement. The encoder uses ResNet-34 optimized with the Mish activation function for deeper feature extraction [38]. Additionally, the Boundary De-Precision module improves the placental boundary delineation and enhances the visualization of blood vessels near the placental border. These advancements highlight the capability of the model to deliver precise and reliable segmentation, even in challenging scenarios such as blurred boundaries owing to PAS. Moreover, the studies also included a diagnostic model based on binary logistic regression that integrates MRI features with clinical factors to predict PAS [39]. This study included 3D MRI images of gestational ages ranging from 28 to 42 weeks, including both normal and PAS cases. This approach underscores the potential of combining clinical insights with imaging data to enhance diagnostic ACC.

Building on the capabilities of RFU-Net, Huang et al. [31] investigated U-Net 3 + [40], an advanced version of the original U-Net designed for segmenting the placenta and uterine cavity from prenatal MRI scans. This model involves 3D MRI images, features intra-skip connections between all decoder layers and employs both max-pooling and bilinear upsampling to effectively integrate features across different scales. The 3D U-Net 3 + architecture includes an encoder-decoder structure for feature capture and localization, full-scale skip connections for enhanced contextual awareness, and hierarchical data integration for learning crucial positional and boundary details. The training set included 101 normal cases, with the model utilizing a hybrid loss function prioritizing placental segmentation and modified to accept 3D MRI inputs, outputting segmentation masks with separate channels for the background, placenta, and uterine cavity. By combining low-level details with high-level positional information, U-Net 3 + significantly outperformed traditional models, showcasing the rapid advancement of deep learning in medical imaging.

Parallel to these, DCO-Net [32] is an innovative adaptation of the traditional U-Net model that stands out for its use of deformable convolutions [41], which adjust both the amplitude of input features and their spatial offsets, adapting to spatial information more effectively for segmentation tasks. The study included gestational ages ranging from 18 to 38 weeks using 2D MRI images. This model incorporates a novel bidirectional O-shaped structural route [42] that enhances its ability to capture the complex variable shapes of the placenta by adding backward skip connections that feed the output of the decoder layers back to the encoder without introducing extra parameters.

Complementing these sophisticated models, the multi-receptive field and mixed attention separation mechanism (MRF-MAS) model [33] introduces a novel placental MRI segmentation framework incorporating a sophisticated encoder-decoder structure utilizing both 2D and 3D MRI scans that address the challenge of segmenting placentas with varying morphologies. It is enriched with two key innovations: a multi-receptive field feature aggregation module and a mixed attention separation mechanism. The former [43] enhances the expression of features across scales using a dual-branch structure with varying convolution kernels to comprehensively capture and integrate diverse placental shapes and sizes. The latter [44] refines the feature representation by separating the input features along the channel direction, applying focused attention to each sub-feature, and reaggregating them to enhance the ACC of segmentation by concentrating on the most relevant features.

In another study, the adoption of the DenseVNet architecture for automatic placental and fetal volume estimation showcased another dimension of deep learning applications [45]. This study utilized 3D MRI scans of infants with gestational ages ranging from 27 to 37 weeks. The volume measurements were derived from the segmented regions within the MRI scans, which involved identifying and outlining the precise anatomical boundaries of the placenta and fetus, where the neural network can differentiate these structures from surrounding tissues by summing up the space occupied by these segmented areas and integrating the segmented slices to compute the total volume in three dimensions. This hybrid model integrates features from DenseNet [46] and volumetric net (V-Net) [47] to enhance the information flow and effectively utilize 3D contextual data.

Finally, Han et al. [34] employed the U-Net CNN to automate the segmentation of placental images from MRI scans. This approach, involving 2D and 3D MRI images, significantly reduces the reliance on manual segmentation, offering a fast, accurate, and clinically relevant method that could potentially streamline and improve the monitoring of fetal health in clinical settings. Table 2 provides a summary of the deep learning algorithms for placental segmentation using MRI, as reviewed in this subsection.

**Table 2:** Comparative analysis of MRI-based deep learning models for placenta segmentation

Model	DC	ACC	IoU	Dataset size	Integration modality
RFU-Net [30]	0.9314	0.9987	0.8620	200 cases (103 normal, 97 PAS) (2D)	U-Net with ResNet34, FMF, RSM, boundary de-precision
DCO-Net [32]	0.8488			871 MRI images (2D)	Deformable convolution, U-shaped architecture, bi-directional O-shaped route
MRI-based diagnostic model [39]				155 cases (93 non-PAS, 62 PAS) (3D)	MRI signs, clinical factors, logistic regression, ROC analysis
3D U-Net 3 + [31]	0.92 (uterine cavity), 0.877 (placenta, sagittal), 0.827 (placenta, axial)			244 axial images (101 normal, 143 suspected PAS) (3D)	3D U-Net 3 + architecture, fully automatic segmentation, end-to-end deep neural networks
MRF-MAS [33]	0.8992		0.8169	390 images (2D/3D)	Multi-receptive field feature structure, mixed attention separation
DenseVNet [45]	Mean 0.925 (validation), 0.928 (testing)			193 MRI scans [104 pregnancies (week 27), 89 pregnancies (week 37)] (3D)	DenseVNet architecture combining DenseNet and V-Net, parametric rectified linear unit activation, dice loss with cross entropy, data augmentation
U-Net [34]		Overall ACC: 0.9868, mean ACC: 0.8671		1110 images (axial, coronal, sagittal) (2D/3D)	U-Net architecture, semantic segmentation, cross-entropy loss

*DC* Dice coefficient, *IoU* Intersection over union

All studies presented advanced methods for placental segmentation and prediction in MRI, each with unique strengths and weaknesses. The RFU-Net focuses on refining segmentation ACC, U-Net 3 + leverages a sophisticated architecture for 3D segmentation, DCO-Net incorporates modulation deformable convolution for enhanced feature extraction, MRF-MAS combines multi-receptive field feature aggregation with mixed attention separation for improved segmentation performance, the MRI-based model integrates clinical and MRI features to predict PAS with high ACC, DenseVNet offers rapid and accurate volume estimation for both placenta and fetus, and U-Net segmentation provides an automatic method with reduced manual intervention.

### Ultrasound-based models

Recent advancements include the use of automated algorithms and machine learning approaches, which have been shown to improve the ACC and reliability of placental segmentation.

Zimmer et al. [24] introduced a multi-task learning methodology that revolves around utilizing knowledge and insights from various related tasks to enhance performance across all tasks [48]. This methodology integrates placental location classification with semantic placenta segmentation using a fully convolutional network tailored for ultrasound imaging. The study utilized several deep learning architectures for placenta segmentation, with gestational ages ranging from 19 to 33 weeks, and 3DUS images. The Enc-Net model [49], an advanced semantic segmentation method that integrates a context encoding module that uses global contextual information to selectively amplify relevant class-dependent feature maps, was employed for placental location classification. The U-Net model, which was refined through hyperparameter optimization and data augmentation, was used for semantic segmentation. This investigation further assessed the effectiveness of multi-task U-Nets (MTU-Net) and transfer-based U-Nets (TU-Net) against a standard U-Net in terms of segmentation ACC.

Building on the concepts of advanced imaging and segmentation, Andreasen et al. [50] introduced a novel multi-center deep learning framework employing a mask R-CNN [51]. This approach combines the advantages of region-based object detection with pixel-wise segmentation to enhance placental classification and segmentation. This study involved 2DUS and 3DUS images across all trimesters and demonstrated robust performance in classifying and segmenting placental images. Moreover, the integration of AI into obstetric ultrasound, as highlighted in ref. [52], has the potential to enhance efficiency, pregnancy outcomes, congenital malformation detection, and adverse pregnancy outcome prediction.

Similarly, according to ref. [53], AI in prenatal fetal ultrasound can hasten examinations and enhance diagnostic ACC by refining standard plane detection, biometric parameter measurement, and disease diagnosis.

Transitioning from multi-view to acoustic enhancements, Hu et al. [54] enhanced placental segmentation in ultrasound images through a CNN that integrated acoustic shadow detection within a U-Net architecture. This approach specifically targets acoustic shadows, sonographic findings characterized by solid structures within the uterus that block sound waves and result in obscured placental boundaries [55]. The study involved 2DUS images at a gestational age range of 8 to 34 weeks. By incorporating a specialized layer that analyzes the acoustic properties to refine the segmentation, the network achieved notable improvements in performance. The inclusion of this layer addresses long-standing challenges in ultrasound image analysis by leveraging advanced deep learning techniques that enhance the encoder-decoder structures and attention mechanisms. Results demonstrated a Dice similarity coefficient of 0.92 ± 0.04 with the shadow detection layer, compared to a slightly lower 0.87 ± 0.04 in images containing acoustic shadows.

To further enhance ultrasound technology, Gleed et al. [56] developed an algorithm for automatic image guidance to assess placental location using ultrasound video sweeps. The model is based on a combination of a U-Net architecture with conditional random fields as recurrent neural network (CRF-RNN) module [57] that refines the segmentation by modeling the spatial relationships between pixels in the segmentation maps, thereby improving the precision of the segmentation boundaries, especially around complex shapes like the placenta. This approach involves ultrasound imaging from a gestational age of 20 to 36 weeks. This demonstrates how integrating temporal and spatial data can significantly advance the precision of medical imaging, particularly in regions with limited access to trained sonographers, notably low- and middle-income countries (LMICs). The U-Net model augmented with a CRF-RNN module demonstrated strong performance in the automatic segmentation of the placenta and maternal bladder in ultrasound images, where the model captured the complex shapes and boundaries necessary for precise medical assessments.

Zimmer et al. [58] efficiently employed CNNs for placental segmentation during late gestation, using multi-view 3DUS images across four models. Model S1 was trained on individual images, whereas Model S2, trained exclusively on fused images, began with a Dice overlap of 0.75 but improved to 0.84 in validation, underscoring its efficacy with extended fields of view. Model S3 enhanced S1 by retraining the fused images. In the last model, S4 focused on refining the segmentation from fused images, highlighting the difficulties in learning from back-projected annotations. These results illustrate the varied capabilities of each CNN model in addressing the challenges of placental imaging during late pregnancy, with clear distinctions in performance, highlighting the importance of model selection based on the specific requirements of the imaging task.

Building on this, Oguz et al. [59] proposed a semi-automated segmentation technique using a multi-atlas label fusion (MALF) approach, a model that uses a series of expert-labeled datasets or atlases, which is a local coordinate representation for understanding anatomical shape variations across clinical populations [60], and helps to guide the segmentation of new images with minimal user intervention. This approach addresses the challenges of accurately segmenting placentas from 3DUS images, such as the high variability and low contrast between the placenta and uterine tissues that are prevalent during early pregnancy.

Furthermore, Perera-Bel et al. [61] implemented a GPU-accelerated random walker (RW) algorithm to enhance the segmentation of the placenta and its vasculature in 3D Doppler ultrasound images with gestational ages ranging from 16 to 36 weeks. This semiautomatic method was used to assist in fetal surgery planning for twin-to-twin transfusion syndrome (TTTS). The RW model’s architecture treats the image segmentation problem through a graph-based approach, where pixels are treated as nodes, and the goal is to determine the ‘label’ of unseeded nodes based on the probabilities derived from seeded nodes [62]. It leveraged both manual and Otsu threshold-based (determining the optimal threshold value for separating an image into foreground and background) initialization for placental and vascular segmentation, accelerating the computation process significantly compared to CPU processing. Table 3 summarizes the deep learning algorithms for placental segmentation with the ultrasound modalities surveyed in this subsection.

**Table 3:** Comparative analysis of ultrasound-based deep learning models for placenta segmentation

Model	DC	ACC	IoU	Dataset size	Integration modality
MTUNet [24]	0.86 (anterior), 0.83 (posterior)	0.91 (classification), 0.86 (anterior segmentation), 0.83 (posterior segmentation)	0.77 (anterior), 0.67 (posterior)	1188 images (classification), 292 images (segmentation) (3D)	Multi-task learning approach combining classification and semantic segmentation, multi-probe image acquisition, image fusion, and image segmentation
Mask R-CNN [50]		0.81 (overall), 0.76 (cross-validation)	0.78 (overall), 0.75 (first trimester), 0.79 (second trimester), 0.73 (third trimester)	7500 images for training, 2130 images with placental regions, 500 images for anterior/posterior classification (2D/3D)	Manual annotations, mask R-CNN model, cross-validation with different datasets
U-Net + CRF-RNN [56]	0.83 ± 0.15 (placenta), 0.66 ± 0.24 (bladder)			2127 frames (10 videos, segmentation), 73,308 frames (135 videos, qualitative assessment)	U-Net with CRF-RNN, U-shaped ultrasound sweep, assistive video overlays
MALF [59]	0.82 ± 0.061 (overall), 0.838 ± 0.063 (anterior), 0.803 ± 0.055 (posterior)			47 ultrasound volumes (28 anterior, 19 posterior) (3D)	MALF, manual annotation of a single central 2D slice, propagation to 3D
GPU-accelerated RW [61]	Placenta/Singleton: average Dice: 0.85, Jaccard: 0.74 Placenta Monochorionic: average Dice: 0.84 Vascular/Singleton: Dice: 0.58 Vascular/Monochorionic: Dice: 0.56			29 pregnancies (5 monochorionic, 24 singleton)	Doppler ultrasound, GPU acceleration, manual initialization with Otsu thresholding
CNN with acoustic shadow detection layer [54]	0.92 ± 0.04 (overall), 0.87 ± 0.04 (acoustic shadow images)			1364 images (2D)	CNN with acoustic shadow detection, diverse dataset from different machines and operators
Multi-view 3D CNN [58]	Dice (S1): 0.80, Dice (S2): 0.75, Dice (S3): 0.86, Dice (S4): 0.70			127 ultrasound (3D)	Multi-view 3DUS imaging, voxel-wise image fusion, 3D CNN-based segmentation

All the research showcases cutting-edge techniques for placenta segmentation and prediction in ultrasound imaging. Multi-task ultrasound segmentation addresses variability and uncertainty while utilizing multi-view acquisition for enhanced segmentation. Multi-center ultrasound segmentation demonstrates robust performance across different populations with high classification ACC. CNN with acoustic shadow detection achieves high ACC with a novel layer to handle acoustic shadows. U-Net + CRF-RNN introduces an assistive overlay to reduce skill requirements for interpretation. Multi-view 3DUS segmentation enables whole placenta segmentation at late gestation with effective multi-view image fusion. Minimally interactive segmentation offers a semiautomated approach with minimal user input and high reliability. The RW algorithm for Doppler ultrasound segmentation provides high ACC and reduced computation time with GPU acceleration. Despite these innovations, all methods face common challenges related to data limitations, preprocessing complexity, and segmentation performance variability.

### Comparative analysis

Benchmarking deep learning models for placental segmentation across MRI and ultrasound modalities offers a perspective on the efficacy and adaptability of each approach in clinical settings. Therefore, high-quality benchmark studies in computational biology should be carefully designed and implemented to provide accurate, unbiased, and informative results [63]​​. This subsection compares the performance (based on ACC, DC, and IoU), technological innovations, and practical usability of various deep learning models. The analysis will delve into the specific strengths and limitations of MRI-based and ultrasound-based models and highlight the versatile applications of the U-Net architecture in these modalities.

### Comparative analysis of MRI-based and ultrasound-based models for placental segmentation

MRI-based models, including 3D U-Net 3 +, typically demonstrate superior segmentation ACC, with DCs often exceeding 0.85. This high level of precision is largely attributed to MRI’s enhanced soft-tissue contrast, which clearly delineates the placenta from the surrounding tissues. MRI models are often at the forefront of advanced computational techniques, notably multi-scale feature aggregation and full-scale connectivity, which allow them to capture detailed anatomical structures and complex interactions within the placenta. However, despite their high ACC, MRI-based models suffer from practical limitations, including high computational demands and complex model architectures, which can hinder their deployment in less technologically advanced clinical environments. Moreover, the intrinsically slow nature and higher costs associated with MRI limit their widespread use, especially in routine prenatal screening.

By contrast, ultrasound-based models are characterized by their real-time imaging capabilities, which are crucial for dynamic clinical environments. Models like the acoustic shadow CNN highlight innovations tailored to handle ultrasound-specific challenges, such as acoustic shadows and speckle noise, which are prevalent in ultrasound imaging but not in MRI. These models are crucial for enhancing the usability of ultrasound, a widely available and relatively low-cost modality, particularly placental segmentation during prenatal checkups in complex scenarios. Although ultrasound models benefit from greater accessibility and real-time data processing, they generally exhibit lower DCs than MRI models. This is partly due to the lower contrast resolution of ultrasound images. In addition, the performance of ultrasound-based models can vary significantly depending on the operator’s skill and the equipment used, which adds another layer of variability that is not as pronounced in MRI-based applications.

**Fig. 4:**
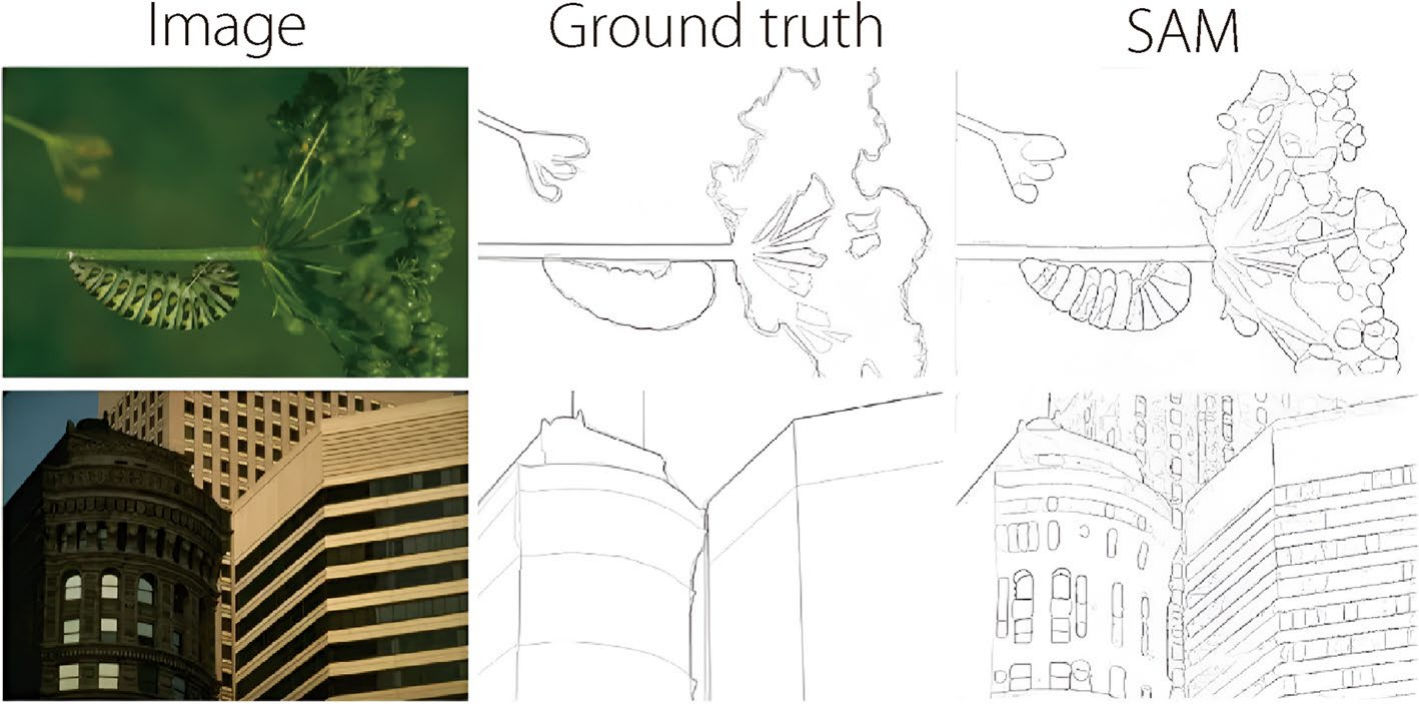
Zero-shot edge prediction with SAM on BSDS500

A notable advancement in image segmentation technology is the segment anything model (SAM), which has the potential to significantly enhance the ACC and efficiency of segmenting complex anatomical structures in the medical field. Although existing literature discusses various deep learning segmentation algorithms applied to MRI and ultrasound imaging, it lacks a comprehensive examination of SAM and its variants, which can achieve high-quality segmentation based on user-defined prompts [64, 65]. The prompt segmentation capability of SAM allows for zero-shot learning, meaning that it can segment objects in images without prior training on specific objects. SAM’s unique architecture includes ‘promptable’ segmentation, where users can provide various types of prompts like points, boxes, masks, or text descriptions (Fig. 4). SAM enables effective segmentation without extensive retraining of specific datasets, which is a crucial feature, given the scarcity of annotated medical data. Its versatility across imaging modalities and ability to automate the segmentation process can streamline clinical workflows and facilitate quantitative analysis, making it a valuable tool for applications ranging from tumor delineation to organ segmentation [66, 67]. Despite its promising potential, SAM has not yet been applied to placental segmentation in MRI or ultrasound. Incorporating SAM into discussions on medical imaging technologies is essential, as it opens new avenues for innovation and addresses the unique challenges faced in medical image analysis [67, 68].

### Innovations and adaptations of U-Net architecture in placental segmentation

Across the studies provided, the U-Net architecture has been employed in various ways to enhance the ACC and efficiency of placenta segmentation in both ultrasound and MRI modalities. Hu et al. [54] used a 2D U-Net with an additional acoustic shadow detection layer to improve segmentation ACC by accounting for ultrasound artifacts. Zimmer et al. [58] utilized a 3D U-Net leveraging multi-view integration to capture the comprehensive spatial features of the placenta. In ref. [24], both 2D and 3D U-Nets were explored, with a focus on multi-task learning to enhance feature extraction by incorporating classification tasks. Saavedra et al. [69] employed a simple yet effective 2D U-Net to identify placenta previa tailored to low-resource settings.

In the MRI modality, Huang et al. [31] utilized a 3D U-Net 3 + with full-scale skip connections to enhance positional and boundary awareness, resulting in highly accurate segmentation. Ma et al. [32] adapted U-Net with deformable convolution layers and bi-directional skip connections to effectively handle the complex edge features of the placenta. A RFU-Net with ResNet34 for feature extraction and a FMF module to capture detailed spatial and contextual information refined the segmentation of blurred boundaries by Li et al. [30]. Finally, Lee et al. [33] combined multi-receptive field feature aggregation with mixed attention mechanisms, enhancing feature detection across various scales and improving segmentation clarity.

Overall, the benchmarking across these studies demonstrated the versatility and adaptability of the U-Net architecture in medical imaging. Each study tailored the U-Net framework to address specific challenges, including handling ultrasound artifacts, integration of multi-view data, or refinement of feature extraction in MRI. This resulted in improved segmentation ACC and robustness, demonstrating the effectiveness of U-Net in diverse medical imaging applications.

In summary, although MRI-based models offer higher ACC and more detailed segmentation capabilities, their practical use is constrained by their cost and complexity. Ultrasound models, although less accurate, provide critical real-time accessible imaging solutions that are adaptable to a wide range of clinical settings. The choice between these modalities and their corresponding deep-learning models depends heavily on specific clinical requirements, available resources, and the desired balance between ACC and accessibility.

## Discussion

Throughout gestation, the placenta undergoes significant morphological changes that influence segmentation outcomes. In early pregnancy, structures are less differentiated and evolve into more complex and vascularized forms as gestation progresses. Advanced deep learning algorithms tailored to specific gestational age ranges have improved segmentation ACC by adapting to these developmental changes. These models effectively address the dynamic nature of placental development using stratified training datasets organized by gestational age.

The integration of deep learning with placental imaging has led to significant advancements in prenatal diagnostics. Benchmark-setting models, for example, RFU-Net and U-Net 3 + for MRI, along with MTUNet and mask R-CNN for ultrasound, have notably improved segmentation ACC. These improvements facilitate early detection of placental abnormalities, enabling better management strategies for maternal and fetal health. However, the high cost and limited availability of advanced imaging technologies and AI models present challenges that must be addressed to make these innovations accessible to a broader population.

Various studies have highlighted the strengths and limitations of MRI and ultrasound modalities for placental segmentation using deep learning techniques. Table 4 provides an overview of the strengths and weaknesses of various MRI and ultrasound segmentation models. The strengths of these models include advanced feature extraction, high adaptability, robustness in handling variability, and field-of-view limitations. However, the weaknesses include challenges including difficulty in locating the placenta in variable images, the need for manual intervention, and dependencies on image quality and operator skill. MRI-based models namely RFU-Net, U-Net 3 +, and DenseVNet have demonstrated high ACC, with performance metrics, for example, DCs ranging from 0.8488 to 0.9314 and mean IoU values of up to 0.8620. These models, which are typically trained on datasets varying in size and gestational age, provide detailed structural information that is beneficial for monitoring complex cases and late pregnancies. Conversely, ultrasound-based models notably acoustic shadow CNN and U-Net + CRF-RNN offer real-time imaging capabilities that are critical for dynamic clinical environments. These models address ultrasound-specific challenges, namely acoustic shadows and speckle noise, thus improving the usability of ultrasound, which is a widely available and relatively low-cost imaging modality. Although these models generally exhibit lower DCs than MRI-based models, they provide practical solutions for routine prenatal check-ups, especially in settings where MRI is not feasible.

**Table 4:** Strengths and weaknesses of various MRI and ultrasound segmentation models

Model	Strength	Weakness
RFU-Net [30]	The multi-scale feature module improves extraction, boundary de-precision highlights potential blood vessels, and the RSM combines deep and shallow features	Difficulty locating the placenta in variable MRI images, boundary detection not applicable for PAS patients, manual intervention required for semi-automatic methods, primarily 2D with needed improvements in 3D segmentation, and complex preprocessing may not suit all clinical settings
DCO-Net [32]	Efficient in automatic segmentation compared to other approaches, with high adaptability and excellent generalization across different placental positions. Outperforms state-of-the-art methods in segmentation ACC and overlap rate with ground truth	Needs more data and improved segmentation techniques, faces training challenges due to large model size and memory constraints, potential data leakage risk with limited data, network under-segments placenta, and requires complex preprocessing
MTUNet [24]	Combines placental location classification and semantic segmentation, addressing variability, annotation uncertainty, and limited field-of-view. Uses multi-view ultrasound for late gestation segmentation, reduces artifacts, extends field-of-view, and shows robustness with consistent performance across different annotations	Performance is influenced by ultrasound image quality and operator dependency. Multi-task learning and multi-view fusion increase computational complexity. Performance may vary due to differences in ultrasound equipment and clinical protocols
MRI-based diagnostic model [39]	Combines clinical factors and MRI features for enhanced diagnostic performance using logistic regression for independent diagnostic models. Validated with an independent set, showing consistent performance, and provides a quantitative reference for individualized treatment and decision-making	MRI methods can’t fully distinguish the range of placental villus invasion. Thin decidual layer and limited T2-weighted imaging resolution hinder sign identification. Observer variability affects diagnostic consistency
3D U-Net 3 + [31]	Full-scale connectivity captures detailed features, achieving high segmentation ACC and effectively handling patient variability with placental complications. Combines advanced techniques like 3D U-Net and data augmentation. Postprocessing refines results by removing small protrusions and filling holes	Network under-segments placenta with large variability. Memory limitations restrict input block size during training. Requires complex preprocessing before training. Over-segmentation of the uterine cavity in some cases
Mask R-CNN [50]	Used multi-hospital data for comprehensive training and validation, outperforming clinicians in placenta delineation. Validated on a Spanish dataset, supporting automated detection of placenta previa and future placental dysfunction research	Diagnostic ACC depends on radiologist experience, causing inconsistencies. Slightly lower ACC in cross-country performance compared to local datasets. Requires more high-quality images of placental tissue. T2-weighted imaging’s limited resolution challenges precise diagnostics
MRF-MAS [33]	Combines channel and spatial attention via MAS for better feature reorganization. Outperforms U-Net, mask RCNN, and Deeplab v3 in placenta segmentation ACC, providing a quantitative reference for precise placenta accreta treatment	Higher training time due to deeper network structure. High computational complexity
U-Net + CRF-RNN [56]	Automatically generates assistive video overlays for placenta location assessment, reducing the skill needed to interpret scans and increasing potential uptake in rural settings and LMICs. The U-Net + CRF-RNN algorithm outperformed the baseline U-Net, especially with complex-shaped placentas	Small sample size, affecting statistical power and validity. Lack of external validation, necessary for confirming generalizability and reliability across different populations and settings
DenseVNet [45]	DenseVNet combines DenseNet and V-Net for enhanced information flow and volumetric analysis, reducing volume estimation time from 60–90 min manually to under 10 sec. It handles variability in placental and fetal volumes across gestational weeks (27 to 37) with high agreement between neural network estimations and manual annotations	High computational complexity and potential risk of overfitting. Needs improvements for segmenting smaller anatomical structures. Additional post-processing steps may be required for refined segmentation
MALF [59]	Semiautomated segmentation for measuring placental volume from 3DUS images with minimal user input. High intraclass correlation (0.86) in test-retest reliability. Outperformed VOCAL software in ACC. Reduces user variability and manual effort	Dependent on initial manual input quality and limited to early pregnancy. Performance varies between anterior and posterior placentas. Sensitive to manual input extent, risking incomplete segmentations. Test-retest reliability shows lower performance than ACC experiments due to a smaller library of 3D atlas images used
GPU-accelerated RW [61]	Near real-time, low inter-user variability, and GPU-accelerated. Detects placenta, umbilical cord insertions, and vasculature from Doppler ultrasound. Rapidly plans fetoscope insertion points for TTTS surgery, improving outcomes	Limited applicability to later pregnancy stages and non-singletons, as the cohort consisted of singleton pregnancies in the first trimester
U-Net [34]	Significantly reduces manual intervention and increases segmentation speed. Uses a comprehensive, clinically significant dataset labeled by experts. Validated with various MRI types (sagittal, cross-sectional, coronal)	Inconsistent results for smaller placenta segments. Potential overfitting with small dataset. Segmentation ACC varies with placental shape complexity
CNN with acoustic shadow detection layer [54]	Outperformed manual segmentation in images with acoustic shadows. Utilized a large, diverse dataset from different machines, operators, and gestational ages. Required no user input for algorithm tuning, enhancing usability in various clinical scenarios	ACC varies with placental shape complexity and acoustic shadows. Dark areas from acoustic shadows can obscure the placenta, causing inaccuracies. Occasionally misidentifies non-placental regions, like the uterine wall or fetal parts, as placenta
Multi-view 3D CNN [58]	Derived placental volumes comparable to MRI. Utilized multi-view image acquisition, image fusion, and segmentation stages. Effective on a dataset of 3DUS images in the last trimester. Enables automatic quantification of placental volume and morphology from ultrasound beyond early stages	Automatic segmentation underestimates placental volume due to poor ultrasound contrast. Manual annotation challenges, especially for thin boundary parts, affect ACC. Smaller training dataset size for multi-view images reduces available training examples, impacting performance

The choice of the imaging modality should be tailored to each clinical scenario. For instance, RFU-Net has demonstrated exceptional ACC (Dice: 0.9314) and precision (ACC: 0.9987) in MRI scans for detecting placental abnormalities, for example, the PAS. Similarly, the mask R-CNN showed robust performance in ultrasound imaging, achieving an overall ACC of 81% across all trimesters, highlighting its utility in routine prenatal care.

In summary, although MRI-based models offer higher ACC and more detailed segmentation capabilities, their practical use is constrained by cost and complexity. Although less accurate, ultrasound models provide critical real-time accessible imaging solutions adaptable to a wide range of clinical settings. Integrating both MRI and ultrasound modalities could potentially enhance segmentation performance by offering the complementary strengths of MRI’s high-resolution anatomical detail and real-time imaging capabilities, which are particularly useful for assessing blood flow. This combined approach could also enable cross-validation of results and improve diagnostic ACC and patient outcomes, especially in prenatal care [70].

**Fig. 5:**
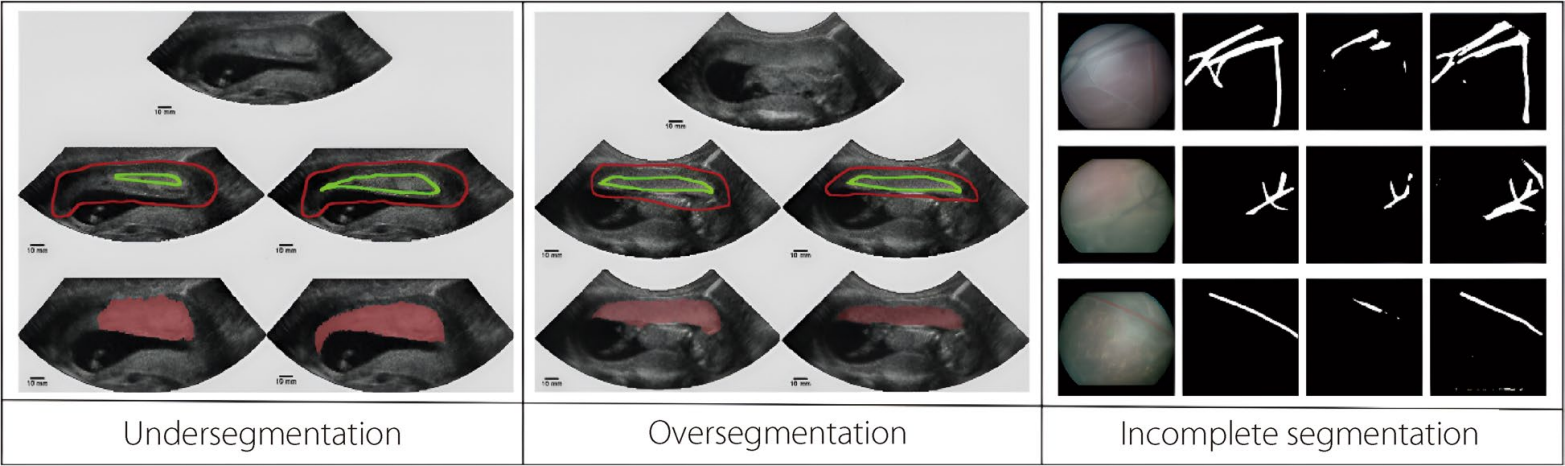
Types of segmentation errors: Undersegmentation, where the user did not treat heterogeneous tissue as placenta compared to correct seeding (Left); Oversegmentation into the fetus compared to correct seeding (Middle); and example of incomplete segmentation of placental vessel segmentation (Right)

Preprocessing techniques play a pivotal role in enhancing the segmentation ACC of placental boundaries in MRI and ultrasound images, particularly in cases of abnormal placentation such as placenta accreta and previa. The primary challenge in placental segmentation arises from the indistinct boundaries between the placenta and surrounding tissues, leading to diagnostic errors [71] and segmentation errors, including inaccurate boundary detection, misidentification of placental subunits, and incomplete segmentation (Fig. 5), which have significant clinical implications. Under-segmentation may underestimate the size of the placenta, whereas over-segmentation can include adjacent organs or tissues, both of which lead to incorrect assessments. Misidentification of structures including the fetal head or bladder can further distort evaluations, and partial segmentation may hinder the accurate assessment of the placenta’s size, shape, and relationship with surrounding structures. Such errors can result in misdiagnosis, suboptimal treatment planning, and an increased risk of complications during pregnancy and delivery, notably postpartum hemorrhage or fetal distress. Addressing these challenges requires advanced preprocessing strategies that enhance contrast, reduce noise, and refine edge detection, thereby improving the quality of the input data for deep learning models. One widely adopted method is contrast-limited adaptive histogram equalization (CLAHE), which has been demonstrated to enhance the visibility of anatomical structures by improving local contrast, thereby aiding in the differentiation of placental tissue from adjacent regions [72]. This technique is particularly beneficial in ultrasound imaging, where speckle noise and low contrast often obscure the placental boundaries, rendering the segmentation unreliable. Additionally, deep learning-based despeckling techniques, namely CNN-based denoising and principal component analysis-based filtering, have shown promise for reducing noise artifacts while preserving structural details, leading to improved segmentation ACC [21].

In addition to contrast enhancement and noise reduction, the incorporation of boundary-aware loss functions in deep learning models has been proven to refine placental segmentation. Standard loss functions often fail to adequately penalize errors at anatomical boundaries, leading to blurred or incomplete segmentation. To address this issue, researchers have explored the integration of dynamic surface and boundary loss functions, which optimize segmentation by explicitly learning tissue interfaces and emphasizing edge preservation [72]. These loss functions have been particularly effective in cases of placenta accreta, in which invasive placental growth results in irregular and diffuse boundaries that are difficult to delineate using conventional segmentation approaches. Additionally, multi-scale feature extraction techniques, for example, pyramid scene parsing modules and pyramid atrous convolution layers, have been used to improve segmentation robustness by capturing both fine-grained anatomical details and larger contextual relationships within an image [73–75]. These techniques enhance the model’s performance in complex imaging scenarios, particularly in MRI, where variations in intensity and texture can complicate placental boundary delineation.

Empirical studies have further supported the efficacy of preprocessing in reducing segmentation errors and improving clinical outcomes. For instance, studies have shown that the application of CLAHE and boundary-aware loss functions in ultrasound image segmentation can lead to an 11% increase in the DC and a 70% reduction in the segmentation errors measured by the Hausdorff distance. Furthermore, hybrid approaches that combine deep learning-based segmentation with atlas-based priors and shape constraints have been explored to further enhance segmentation ACC by incorporating prior anatomical knowledge into the learning process of the model. These methods ensure that the segmented placenta adheres to realistic morphological constraints, mitigating errors caused by blurred boundaries and misclassification of surrounding structures.

Stochastic resonance (SR) is a promising approach to overcome the inherent limitations of ultrasound and MRI in placental imaging. Despite their advantages, both imaging modalities face considerable challenges; ultrasound images are often hindered by low resolution, speckle noise, and shadowing effects, which obscure critical anatomical details [76, 77]. However, MRI can suffer from motion artifacts due to fetal movements and maternal breathing [78], along with varying signal-to-noise ratios that degrade image quality [79]. These imaging constraints make accurate segmentation of the placenta from the surrounding tissues particularly difficult. To address these challenges, enhanced imaging techniques, for example, diffusion-weighted imaging, advanced computational methods, 3D reconstruction [80] and machine learning-based texture analysis [81] are being explored. Recognizing features notably uterine bulging or abnormal vascularization, alongside traditional boundary assessments, have been shown to improve diagnostic ACC in conditions such as PAS [82].

SR, a nonlinear phenomenon in which moderate levels of noise enhance the detection of weak signals, has been demonstrated to be a useful technique for improving image quality in medical imaging. In ultrasound imaging, SR can mitigate the effects of speckle noise by constructively leveraging noise to enhance low-contrast images, thereby making the placental boundaries more distinct. Similarly, SR techniques can be applied to MRI to reduce motion-induced artifacts by stabilizing the signals and improving feature extraction of the placenta [83]. The combination of SR with advanced image processing techniques, namely principal component analysis, can further refine segmentation ACC by enhancing contrast and suppressing noise in both imaging modalities [84]. Previous studies have shown that SR-based denoising using the maximal overlap discrete wavelet transform effectively enhances image clarity by optimizing signal-to-noise ratios, a technique that has proven beneficial in brain aneurysm segmentation and other medical applications [85].

Although ultrasound remains the primary screening tool owing to its accessibility, MRI serves as a complementary modality, particularly when ultrasound findings are inconclusive [86, 87]. Continued advancements in imaging technologies and methodologies are crucial for overcoming segmentation challenges in placental imaging. The integration of SR-enhanced imaging with state-of-the-art computational techniques has significant potential to improve the detection and diagnosis of placental abnormalities.

Although ultrasound remains the primary screening tool owing to its accessibility, MRI serves as a complementary modality, especially when ultrasound findings are inconclusive [86, 87]. Continuous advancements in imaging technologies and methodologies are essential for overcoming segmentation challenges and enhancing the diagnosis of abnormal placentation.

In conclusion, while both MRI and ultrasound have unique and complementary roles in fetal imaging, the choice of modality often depends on the specific diagnostic requirements, the area of the fetus being examined, and the clinical scenario. Their combined application can optimize diagnostic effectiveness, thus enhancing prenatal care and management. Enhanced imaging technologies, such as 3DUS and AI-enhanced MRI, continue to revolutionize fetal medicine by improving the detection rates of congenital anomalies, although they remain limited by availability and cost considerations [88].

## Conclusions

The application of deep learning techniques in placental segmentation has revolutionized prenatal diagnostics, significantly enhancing ACC and efficiency. When integrated with AI, both MRI and ultrasound offer complementary benefits that bolster diagnostic capabilities. This comprehensive review covers various deep learning models and underscores their potential to address the complexities of placental imaging, including issues related to fetal position variations, dynamic placental development, and varying image quality. This review predominantly focuses on recent studies from 2019 to 2024, with an emphasis on the latest advancements from 2023 to 2024.

Our review highlights several advanced models, such as RFU-Net, U-Net 3 +, and DenseVNet for MRI, as well as MTUNet and mask R-CNN for ultrasound. These models have established new benchmarks for segmentation ACC and have facilitated the early detection of placental abnormalities, thereby contributing to improved maternal and fetal health management.

However, the high costs and limited availability of advanced imaging technologies present significant challenges. To address these issues, future studies should focus on developing adaptable and cost-effective models that can be seamlessly integrated into clinical workflows to ensure broader accessibility. This includes leveraging both MRI and ultrasound modalities to provide a comprehensive evaluation of the placenta, thereby enhancing diagnostic ACC and improving patient outcomes.

Moreover, the evolution of AI in medical imaging promises further advancements in maternal and fetal health. By addressing challenges, for example, high costs, limited availability, and the need for extensive training datasets, researchers can make advanced prenatal care more accessible to a broader population. Emphasis should also be placed on ethical considerations, namely patient privacy and the interpretability of AI models, to ensure that the deployment of these technologies is both responsible and effective. Benchmarking in Lean healthcare improves system effectiveness by spreading best practices and facilitating performance comparisons at various levels [89]​​. Similarly, benchmarking can identify shortcomings and justify reforms, thereby enhancing the efficiency and effectiveness of healthcare systems [90]​​.

This review provides an overview of recent advancements in placental segmentation using deep learning with ultrasound and MRI. However, it is important to acknowledge the limitations of this study. First, the focus on the literature from 2019 to 2024 may overlook earlier foundational studies that could provide valuable context for understanding the evolution of segmentation techniques. Second, although the strengths and weaknesses of different modalities and techniques are discussed, a comprehensive quantitative comparison across studies is challenging, owing to variations in datasets, evaluation metrics, and experimental setups. Finally, the scope of the review was constrained to existing literature, which may not capture emerging trends or novel approaches that have yet to be published, thereby limiting its forward-looking perspective on future research directions in placenta segmentation.

In summary, the integration of deep learning into placental segmentation represents a significant advancement in prenatal diagnostics. This review synthesizes recent research, expands knowledge in this innovative area, and highlights the transformative impact of deep learning. Future work will focus on enhancing model generalizability, reducing data requirements, and ensuring robust performance in diverse clinical settings. The continued advancement of AI-driven medical imaging holds great promise for improving diagnostic strategies and health outcomes in prenatal care.

## Data Availability

Not applicable.

## Abbreviations

MRI: Magnetic resonance imaging
PAS: Placenta accreta spectrum
HPC: High-performance computing
SoC: System-on-chip
CUDA: Compute unified device architecture
FPGA: Field-programmable gate array
CNN: Convolutional neural network
GPU: Graphics processing unit
AI: Artificial intelligence
RFU-Net: Refinement fusion U-Net
FMF: Fusion multi-scale feature
RSM: Refinement segmentation module
MTU-Net: Multi-task U-Net
TU-Net: Transfer-based U-Net
CRF-RNN: Conditional random fields as recurrent neural network
LMICs: Low- and middle-income countries
MALF: Multi-atlas label fusion
3DUS: Three-dimensional ultrasound
2DUS: Two-dimensional ultrasound
RW: Random walker
TTTS: Twin-to-twin transfusion syndrome
SAM: Segment anything model
CLAHE: Contrast-limited adaptive histogram equalization
ACC: Accuracy
DC: Dice coefficient
SR: Stochastic resonance
IoU: Intersection over union
V-Net: Volumetric net
MRF-MAS: Multi-receptive field and mixed attention separation mechanism
